# Failure of BACTEC™ MGIT 960™ to detect *Mycobacterium tuberculosis* complex within a 42-day incubation period

**DOI:** 10.4102/ajlm.v6i1.537

**Published:** 2017-04-26

**Authors:** Sharana Mahomed, Nomonde R. Dlamini-Mvelase, Moses Dlamini, Koleka Mlisana

**Affiliations:** 1 National Health Laboratory Services, Inkosi Albert Luthuli Central Hospital, Durban, KwaZulu Natal, South Africa; 2 Department of Medical Microbiology, University of KwaZulu-Natal, Durban, South Africa

## Abstract

For the optimal recovery of *Mycobacterium tuberculosis* from the BACTEC™ Mycobacterium Growth Indicator Tube 960™ system, an incubation period of 42–56 days is recommended by the manufacturer. Due to logistical reasons, it is common practice to follow an incubation period of 42 days. We undertook a retrospective study to document positive Mycobacterium Growth Indicator Tube cultures beyond the 42-day incubation period. In total, 98/110 (89%) were positive for *M. tuberculosis* complex. This alerted us to *M. tuberculosis* growth detection failure at 42 days.

## Introduction

Tuberculosis remains an important cause of morbidity and mortality, especially in developing countries. Recent molecular methods, in particular the Cepheid GeneXpert™, have revolutionised the rapid diagnosis of tuberculosis due to their high sensitivity, even in smear-negative sputum samples. Although culture-based methods take longer, they remain an integral part of the laboratory diagnosis of tuberculosis. The Clinical and Laboratory Standards Institute recommends that both solid- and liquid-based culture methods be used to maximise the recovery of *Mycobacterium tuberculosis*.^1^

In comparison to solid-based culture methods, liquid-based culture methods have the advantage of providing more rapid results and, thus, are commonly utilised in tuberculosis diagnostic laboratories. Previously, the BACTEC™ 460TB™ system (Becton Dickinson Diagnostic Systems, Sparks, Maryland, United States) was widely used for the diagnosis of tuberculosis, but due to the disadvantage of containing a radioactive-labelled substrate, it was replaced by the non-radiometric BACTEC™ Mycobacterium Growth Indicator Tube (MGIT) 960™ system. This system can be used for the detection of mycobacteria in all types of clinical specimens, except blood. It contains a liquid culture medium (modified Middlebrook 7H9 broth), a growth supplement and the antimicrobials polymyxin, amphotericin, nalidixic acid, trimethoprim and azlocillin, to prevent growth of contaminants. A fluorescent compound is embedded in silicone at the bottom of the 16x100 mm tube. The fluorescence indicator is initially quenched by large amounts of dissolved oxygen. As organisms grow and respire, they consume the oxygen, which allows the compound to fluoresce.^2^

For the optimal recovery of *M. tuberculosis*, an incubation period of 42–56 days is recommended by the manufacturer. In addition, the manufacturer recommends that a visual check be performed on all ‘instrument negative’ MGIT tubes. If the tube is shown to contain small granular clumps or appears non-homogeneously turbid, an acid-fast bacilli (AFB) stain should be performed. If the AFB smear is positive, then the tube should be regarded as a presumptive positive. Although these recommendations are clearly stated by the manufacturer, due to logistical and practical reasons, it is common practice to follow an incubation period of 42 days in most tuberculosis laboratories. Studies have illustrated that the sensitivity of the system increases by prolonging the incubation period beyond 42 days.^3^

In our laboratory, which is located in a busy tuberculosis referral hospital and processes 12 000 MGIT tubes a month, we follow an incubation period of 42 days. MGIT tubes are incubated until the instrument flags them positive. After 42 days, the instrument flags the tubes negative if there is no growth. All MGIT tubes that are negative at 42 days are removed from the system. However, it was brought to our attention that MGIT tubes were, in fact being left in the system beyond 42 days during public holidays and over weekends. More importantly, it was noted that some of these MGIT tubes flagged positive after 42 days. Ziehl-Neelson (ZN) staining revealed the presence of AFB with and without cording. These AFB-positive MGIT tubes with positive cording were thereafter sent for a *MTBDR+* line probe assay (Hain Lifescience GmbH, Nehren, Germany), which confirmed the presence of *M. tuberculosis* complex. This alerted us to the possibility that the system failed to detect the growth of *M. tuberculosis* at 42 days. We therefore undertook a study to document our experience regarding MGIT tube cultures that became positive beyond the usual 42-day incubation period.

## Research method and design

### Ethical considerations

Blanket ethical approval for this laboratory-based study was obtained from the Biomedical Research Ethics Committee, University of KwaZulu-Natal, South Africa. The confidentiality of the data was maintained throughout the study.

### Study setting

This study was conducted at the National Health Laboratory Services, Provincial TB Reference laboratory, based at the Inkosi Albert Luthuli Central Hospital. This laboratory currently performs all culture and culture-based phenotypic drug susceptibility testing for the province of KwaZulu-Natal, South Africa.

A retrospective study for the period 01 January 2014 to 31 March 2015 (15 months) was performed. BD EpiCenter™ (Becton, Dickinson and Company, Franklin Lakes, New Jersey, United States) software was utilised to specifically look at all MGIT tubes that flagged positive after the period of 42 days. This software provides advanced data management for all BD Microbiology systems. The basic configuration of the software provides all the communication and reporting capabilities optimised for supporting BD BACTEC™ and BD BACTEC MGIT™ systems.

ZN staining was performed on all positive MGIT tubes to determine whether AFB was present and to document cording characteristics. MGIT tubes were presumed to be positive for *M. tuberculosis* if: (1) AFB were present (straight or slightly curved rods), (2) a beaded appearance was observed due to cording characteristics, and (3) staining was pink on a blue background. In order to discriminate between *M. tuberculosis* and mycobacteria other than tuberculosis, testing for antigens to the MPT64 protein (MPT) and line-probe hybridisation assays were performed on all isolates that were AFB-positive. We used the SD BIOLINE TB Ag MPT 64 Rapid® (Standard Diagnostics, Seoul, Korea) immunochromatographic commercial assay, which uses monoclonal antibodies against the MPT64 antigen for confirmation of *M. tuberculosis* isolates. As part of the routine workflow, all *M. tuberculosis*-positive isolates were subjected to line probe assay testing (Hain Lifescience GmbH, Nehren, Germany) for anti-tuberculosis drug susceptibility, as per standard operating procedures.

Late contaminants were defined as isolates that flagged positive on the MGIT system but were AFB-negative with ZN staining. Mycobacteria other than tuberculosis were identified as isolates that were AFB-positive with ZN staining but MPT/line-probe assay-negative. Isolates that were AFB-positive, exhibited cording and were MPT-positive were considered to be positive for *M. tuberculosis* complex.

## Results

A total of 20 914 MGIT tubes flagged positive during this period. Of these, 159 flagged positive after 42 days (0.8%). A total of 49/159 (31%) were negative on ZN staining and were regarded as late contaminants, and 110/159 (69%) were positive on ZN staining. A total of 12/110 AFB-positive isolates (11%) exhibited no cording, underwent MPT antigen testing, were found to be negative, and were thus identified as mycobacteria other than tuberculosis. A total of 98/110 (89%) exhibited cording, were MPT-positive, and were therefore identified as positive for *M. tuberculosis* complex. Only 78/98 isolates were submitted for line probe assay testing, which further identified all 78 isolates as *M. tuberculosis* complex. Of these 78 isolates, 37/78 (47.4%) were sensitive to both isoniazid and rifampicin, 7/78 (9%) were resistant only to rifampicin, 33/78 (42.3%) were resistant to both isoniazid and rifampicin (i.e., multi-drug resistant) and 1/78 (1.3%) was identified as extensively drug resistant.

## Discussion

A number of studies have reported that the BACTEC MGIT 960 system is efficient, rapid, reliable, safe and fully automated, with the added advantage of continuous monitoring of the culture tubes.^3,4,5,6,7,8,9^ Although most studies have confirmed that the BACTEC MGIT 960 has a high sensitivity for recovery of mycobacteria, it has been reported that the instrument detection system may occasionally fail to detect mycobacterial growth at the end of a 42-day incubation period.^4,5,10^ This has been observed with mycobacteria other than tuberculosis isolates, with *M. xenopi* being highlighted specifically.^4,10,11^ This failure to detect growth at 42 days was attributed to the granular growth pattern of the organism, which created less surface contact, keeping oxygen consumption below the detection threshold. The small bacterial load, slower metabolism, and biochemical and thermophilic characteristics of these isolates were also considered as possible contributing factors.^10^ Failure of the BACTEC MGIT 960 to specifically detect *M. tuberculosis* complex isolates within a 42-day incubation period has not previously been reported.

The findings of our study are important in that of the 20 914 MGITs processed by our laboratory, 159 (0.8%) were flagged positive *after* 42 days and were identified purely by a failure to rigidly follow the laboratory protocol. Although this represents a small percentage overall, 110/159 MGITs were AFB-positive. More importantly, 89% of these (98/110) were subsequently identified as positive for *M. tuberculosis* complex, of which 52.6% (41/78) represented drug-resistant *M. tuberculosis*.

Time to detection of positive growth depends on several factors. The number of viable AFB inoculated into a MGIT tube, the type of species of mycobacteria, the specimen type and the treatment status of the patient must all be taken into consideration. In order to avoid false negatives, the manufacturer refers to troubleshooting procedures in the manual.^12^ Incubation temperatures, decontamination procedures, centrifugation, the use of antibiotics (polymyxin, amphotericin, nalidixic acid, trimethoprim and azlocillin) and procedure checks need to be rigorously carried out in order to avoid missed detection. This study therefore highlights the importance of following laboratory protocols and manufacturer guidelines.

### Limitations

This study was purely laboratory-based and all data were accessed via the MGIT and EpiCenter software. We did not have access to patient information for the resistant samples; thus, the treatment status of these patients, which could possibly be related to the delayed positivity, could not be ascertained.

### Conclusion

These findings present a challenge for routine, clinical *M. tuberculosis* laboratories regarding the dogma of incubating tuberculosis cultures for 42 days only. A possible solution to avoid missing *M. tuberculosis* complex, including resistant strains, would be to prolong the incubation period beyond 42 days. However, this would prove impractical in busy tuberculosis laboratories, due to space constraints and the increased number of MGIT tubes that would be required. A more practical option would be to routinely visually examine all negative MGIT tubes at 42 days as recommended by the manufacturer. If colony-like clumps are visible at the bottom of the tube, then these MGITs should be further analysed. An AFB smear should first be performed and, if found to be AFB-positive, the sample should be reported as presumptive positive and sent for further testing according to the laboratory’s protocol. Troubleshooting guidelines, quality control for the reagents and products used in the isolation, as well as the actual test procedures, are critically important for mycobacteriology laboratories. This study serves to highlight the possibility of missed *M. tuberculosis* due to growth detection failure. If there is a high suspicion of tuberculosis and the result is negative, this must be communicated to the laboratory. It is thus imperative to maintain good communication between laboratories and clinicians. Good history taking and clinical judgement guide the laboratory and thus cannot be over-emphasised.
